# Experiences and Views of Older Adults of South Asian, Black African, and Caribbean Backgrounds About the Digitalization of Primary Care Services Since the COVID-19 Pandemic: Qualitative Focus Group Study

**DOI:** 10.2196/57580

**Published:** 2024-12-18

**Authors:** Nisar Ahmed, Alex Hall, Brenda Poku, Jane McDermott, Jayne Astbury, Chris Todd

**Affiliations:** 1 National Institute for Health and Care Research (NIHR) Policy Research Unit in Older People and Frailty / Healthy Ageing, School of Health Sciences, Faculty of Biology, Medicine and Health The University of Manchester Manchester United Kingdom; 2 School of Sociology and Social Policy, University of Nottingham The University of Nottingham Nottingham United Kingdom

**Keywords:** digital health and primary care services, digital exclusion, digital divide, health inequalities, older adults, South Asian, Black African, Caribbean, COVID-19 pandemic, qualitative focus group study

## Abstract

**Background:**

The COVID-19 pandemic from 2020 to 2022 prompted governments worldwide to enforce lockdowns and social restrictions, alongside the rapid adoption of digital health and care services. However, there are concerns about the potential exclusion of older adults, who face barriers to digital inclusion, such as age, socioeconomic status, literacy level, and ethnicity.

**Objective:**

This study aims to explore the experiences of older adults from the 3 largest minoritized ethnic groups in England and Wales—people of South Asian, Black African, and Caribbean backgrounds—in the use of digitalized primary care services since the beginning of the COVID-19 pandemic.

**Methods:**

In total, 27 individuals participated in 4 focus groups (April and May 2023) either in person or via online videoconferencing. Patient and public involvement and engagement were sought through collaboration with community organizations for focus group recruitment and feedback on the topic guide. Data were analyzed using framework analysis.

**Results:**

This paper summarizes the perspectives of 27 older adults from these 3 minoritized ethnic groups and identifies four key themes: (1) service accessibility through digital health (participants faced difficulties accessing digital health care services through online platforms, primarily due to language barriers and limited digital skills, with reliance on younger family members or community organizations for assistance; the lack of digital literacy among older community members was a prominent concern, and digital health care services were felt to be tailored for English speakers, with minimal consultation during the development phase), (2) importance of face-to-face (in-person) appointments for patient-clinician interactions (in-person appointments were strongly preferred, emphasizing the value of physical interaction and connection with health care professionals; video consultations were seen as an acceptable alternative), (3) stressors caused by the shift to remote access (the transition to remote digital access caused stress, fear, and anxiety; participants felt that digital health solutions were imposed without sufficient explanation or consent; and Black African and Caribbean participants reported experiences of racial discrimination within the health care system), and (4) digital solutions (evaluating technology acceptance; participants acknowledged the importance of digitalization but cautioned against viewing it as a one-size-fits-all solution; they advocated for offline alternatives and a hybrid approach, emphasizing the need for choice and a well-staffed clinical workforce).

**Conclusions:**

Digital health initiatives should address the digital divide, health inequalities, and the specific challenges faced by older adults, particularly those from minoritized ethnic backgrounds, ensuring accessibility, choice, and privacy. Overcoming language barriers involves more than mere translation. Maintaining in-person options for consultations, addressing sensitive issues, and implementing support systems at the practice level to support those struggling to access services are vital. This study recommends that policy makers ensure the inclusivity of older adults from diverse backgrounds in the design and implementation of digital health and social care services.

## Introduction

### Background

For around 2 decades, the World Health Organization has been encouraging the use of digital technologies to improve health and social care services [1]. The COVID-19 pandemic from 2020 to 2022 led governments worldwide to mandate national lockdowns and social restrictions, often accompanied by rapid adoption and implementation of digital and remote modes of access to and delivery of health and care services [2,3]. In the United Kingdom (the setting for this study), recent and postpandemic health care policy and strategy focus on a “digital first” vision that prioritizes digital access to services [4,5], facilitated primarily by the National Health Service (NHS) app as the “front door” to health services, greater use of electronic care records, and digital self-management of long-term conditions [6]. Although such approaches may facilitate access to essential services and offer numerous advantages for patients, there are concerns about the potential exclusion of social groups considered disadvantaged. Digital exclusion (where people or groups in society are “unable to exploit the benefits from technologies”) [7] involves factors such as age; socioeconomic status; education level; ethnicity; limited infrastructure in rural or deprived areas; and people’s digital skills, motivation, and health literacy [8,9]. Relationships between these factors can be complex. Older adults are the least likely group to access and use the internet, but statistics on access and use do not show the full picture, and further work is required to explore the nuanced interactions between age and other factors that may influence the motivation to use digital technologies for health [7,10].

A growing body of literature highlights a discrepancy between policy makers’ visions of efficient, safe, and accessible digital, remote access health services and empirical work showing that the reality is much more complex, influenced by numerous factors such as technological design and functionality; physical environments; and patient and health care professional views, expectations, and experiences [11]. Challenges and barriers to remote digital health care access that particularly affect older adults and that have potentially been exacerbated during the COVID-19 pandemic have been outlined in several studies [12-16]. Mubarak and Suomi [13] underscore the pressing issue of digital exclusion among older adults, particularly in high-income countries where the challenges of adopting the digital revolution are prominent, potentially impacting the provision of health care and service delivery. Basic technology is now widely available in high-income countries, but there still remains a digital divide because not everyone is technologically proficient. For example, in New Zealand, certain communities considered marginalized continue to experience digital exclusion despite government initiatives that have provided technological infrastructure and skills training [17]. The digital divide highlights access gaps, while digital exclusion addresses the specific reasons and impacts of being left out. In emerging and developing nations such as India, there may be a high percentage of computer and internet users, but some social groups remain behind as only the wealthy are able to bridge this gap [18]. Barriers such as inadequate equipment, social isolation, insufficient computer skills and difficulty learning such skills, lack of trust in online information, or health problems can contribute to these gaps. The COVID-19 pandemic increased combined feelings of social and digital exclusion among older people, highlighting the urgent need for specialized technology access, training, and continuous assistance [16]. During the COVID-19 pandemic, older people in many countries were advised to isolate for extended periods during lockdowns, and switches to digital modes of service access and delivery meant that they were disproportionately affected in both their social lives and in health care access and outcomes [15]. Even when they desire to learn about digital technologies, older people are likely to need assistance and support [19].

A synthesis of review-level evidence published before the COVID-19 pandemic on the impact of digital technologies on access to health and social care services for older adults (aged >65 years) concluded that the evidence was unclear, of low quality, and insufficient to support the effectiveness of these technologies in improving older adults' access to services [20,21]. A scoping review of 22 reviews that mapped disparities in digital health technology engagement, use, and access across equity domains in the World Health Organization’s European region found clear, consistent disparities in digital health care access and use based on age, race, language, education, socioeconomic status, and urban residence [22]. The reviews showed that White, English-speaking people without impairments and younger, better educated, affluent people living in urban areas had the highest use of digital health technologies. There was inconclusive evidence of variations in the use of digital technologies based on factors such as occupation, gender or sex, and disability, and there was no clear evidence regarding disparities in engagement with digital technologies [22]. A systematic review of evidence on inequalities in remote consultations in general practice, published before the COVID-19 pandemic and covering all patient age groups, found that telephone consultations were more commonly used by younger individuals, the older adults, and nonimmigrants, while internet-based consultations were primarily used by younger people [23]. Other work has highlighted the potential impact of ethnicity on digital exclusion. Longitudinal quantitative work investigating the impact of the COVID-19 pandemic on primary care consultations for adults with multiple health conditions suggests that there were ethnic inequalities in service access, as people from Black and Asian backgrounds saw a higher reduction in face-to-face consultations [24]. A recent scoping review on the use of digital health by people from South Asian communities emphasizes that people from this ethnic group can struggle to navigate health care systems digitally and highlights the need for culturally relevant interventions and digital skills development [25]. In a mixed methods study undertaken in the United States [26], researchers explored how older adults from diverse ethnic backgrounds engage with digital health information (DHI). The study revealed significant disparities in computer ownership, internet access, and DHI use among ethnic groups, with factors such as older age, lower income, lower education, and minority status predicting limited DHI use. Focus groups provided nuanced insights into the unique experiences and preferences of different ethnic groups, such as frustration with DHI access among African Americans, skepticism about information sources among Hispanic Americans, and active evaluation of DHI websites among European Americans. The study highlights the urgent need to address these disparities to ensure equitable access to digital health resources for minoritized older adults considered economically disadvantaged [26]. While this study provides valuable insights into the experiences of older adults from minoritized ethnic groups with digital health, its focus is on accessing health information rather than on interaction with health services. There are also limitations in transferring study findings from the US health system and cultural and socioeconomic contexts to those of the United Kingdom.

Recent work in the United Kingdom exploring the safety of remote primary care consultations found that patient safety can be compromised by several key factors, such as inappropriate consultation modality, poor rapport building, and inadequate attention to the social circumstances of the patient [27]. Qualitative research conducted before the COVID-19 pandemic (with White British participants mainly aged <60 years) highlighted that while participants felt that online consultation tools could increase access for certain groups (eg, people who have difficulties communicating verbally), they also struggled with the laborious nature of structured questionnaires and with articulating their problems independently [28]. Other work exploring the impact of COVID-19 pandemic–driven digitalization with migrants (aged <50 years) found that digitalization has aggravated existing inequalities in access to health care because of challenges in digital literacy and access to technology, which are augmented by language barriers. In addition, recent non–peer-reviewed reports have suggested that health inequalities have been widened because of the COVID-19 pandemic, driven at least in part by the digital divide [29-32]. Some qualitative studies have highlighted the challenges faced by digitally excluded individuals in accessing health services during the COVID-19 pandemic, including language barriers for those with limited English proficiency [33-37].

While there is a body of research examining the role of personal characteristics, including age and ethnicity, in health inequalities and digital exclusion, specific gaps remain. Existing studies have provided valuable insights into these issues, including work produced in the United Kingdom. However, there is a need for more nuanced and context-specific research that explores the experiences of older adults from minoritized ethnic groups within the United Kingdom’s unique health care system and policy environment. Overall, there is a dearth of qualitative evidence in the UK peer-reviewed literature that focuses specifically on the perceptions and experiences of older adults from minoritized ethnic groups on the digitalization of health and care services since the start of the COVID-19 pandemic. The lack of qualitative research on the perspectives of older adults from minoritized ethnic groups is an important gap because engagement and use of digital interventions are influenced by cultural differences and perspectives [7]. There is a need for much more detailed focus on the impacts of COVID-19 pandemic–driven digitalization of health and care services and potential health inequalities among older people from minoritized ethnic groups. It is important to understand how digital access to health and care services is experienced by diverse groups of older people across society to address digital exclusion through targeted interventions, cultural considerations, and ongoing support to ensure equitable digital access and engagement. Our study focuses on South Asian, Black African, and Caribbean older adults in the United Kingdom to provide nuanced insights into their unique barriers and facilitators regarding digital health services. This study addresses critical gaps, particularly the lack of qualitative studies on older adults from minoritized ethnic groups regarding digital health services after the COVID-19 pandemic. Understanding their experiences is vital for developing targeted interventions and ensuring equitable access to digital resources.

This paper reports on a qualitative focus group study that aimed to advance the understanding of how older adults from 3 minoritized ethnic groups have experienced digitalized health and care services since the beginning of the COVID-19 pandemic in the United Kingdom. We were interested in learning what has worked well and what has not, with a specific lens on the digital divide and health inequalities. The study specifically focuses on experiences of older adults from South Asian, Black African, and Caribbean groups, as they are the 3 largest minoritized ethnic groups in England and Wales [29]. This study focuses on older adults from South Asian, Black African, and Caribbean backgrounds who are at risk of digital exclusion due to barriers such as language and cultural differences.

### Objectives

The research objectives of this study were as follows:

- Explore the general experiences of older adults from South Asian, Black African, and Caribbean backgrounds in accessing or using digitalized primary care services such as general practitioners (GPs), pharmacies, dentists, and optician services in England.
- Investigate the views and perceptions of older adults from these backgrounds on key aspects of their experiences with digitalized primary care services, including health care access, social support systems, and cultural influences, in order to delve deeper into the factors influencing digital engagement, with a particular focus on health inequalities.
- Examine the expectations of older adults from these groups regarding future digital primary care services.

## Methods

### Study Design, Participant Criteria, and Recruitment

The study adopted a qualitative design using focus groups. Participants were eligible to take part if they were aged >65 years and from either a South Asian, Black African, or Caribbean background. There were no requirements for English language proficiency, as we intended to facilitate interpreter support, if necessary; community group members who arranged the focus groups and facilitators were also available during the sessions to help with translation. We viewed digital engagement as being on a spectrum ranging from people who are highly engaged and digitally literate to those who are digitally excluded. Digitally excluded individuals lack access to and proficiency in using digital technologies, limiting their ability to participate completely in the digital society. This can be a function of a range of material and psychosocial factors, such as skills, self-confidence, appropriate technological resources, and available support [38]. Exploration of these factors is crucial to understand people’s engagement and experiences with digital technologies. We applied a combination of purposive and convenience sampling approaches to attempt to identify and recruit participants across the spectrum of digital engagement.

To recruit digitally engaged older adults, the study was advertised via the Valuing Our Intellectual Capital and Experience platform, a national community of people interested in health and care research for older people. Those interested in taking part were emailed further information about the study and a consent form. To recruit older adults who are less digitally engaged or digitally excluded, a participatory methodology was used, which is particularly pertinent for working with groups considered potentially socially disadvantaged [39]. This involved continued engagement with an established public and community advisory group of members within the Greater Manchester Older People’s Network (GMOPN) and the use of existing links with the Caribbean and African Health Network (CAHN; a national organization based in Manchester). Through these contacts, the research team was connected to a South Asian community group via the GMOPN, where community leaders were asked to share written information in the form of printed sheets in appropriate languages about the study among their communities and to coordinate expressions of interest, which were then followed up by the research team. The CAHN recruited participants directly on behalf of the research team in accordance with our inclusion criteria through advertising the opportunity to take part through their networks and via follow-up telephone conversations to confirm involvement.

Participants’ self-perceived levels of digital engagement and confidence were assessed through open-ended questions during the focus group discussions. Participants were invited to share their experiences regarding their use of digital technologies, including the frequency of use, comfort level with various digital tools, and perceived barriers to engagement. This qualitative approach enabled a nuanced understanding of their self-assessment of digital engagement and confidence. In addition, participants were recruited through community groups to engage individuals who might be at a higher risk of “digital exclusion.”

### Data Collection

Participants were invited to attend a focus group to discuss their views and experiences of digitalized primary care health services. Focus group methods were used as they allow researchers to collect diverse views from multiple people at once. Crucially, we felt that the element of group dynamics afforded by this approach and the chance for participants to discuss their perspectives with their peers as well as with the researchers would offer greater insight into cultural perspectives on the digitalization of health and care services than one-to-one interviews [40].

Topic guides for focus groups were prepared (Multimedia Appendix 1); these were structured outlines or lists of key topics, questions, and prompts that guided the discussion during a focus group session. These guides helped ensure that the conversation remained focused on the research objectives. The topics included in the guide covered the main themes the researchers wanted to explore.

The discussion focused on experiences of accessing primary care services such as GPs, pharmacies, dentists, and optician services via digital technologies and views and expectations about the future of primary care services and digital health technologies. Participants self-identified their ethnic group.

Focus groups were arranged based on participants’ ethnic backgrounds, with separate groups for those from South Asian backgrounds and those from Black African or Caribbean backgrounds. Participants from Black African and Caribbean backgrounds were grouped together in focus groups due to shared sociocultural experiences and challenges regarding health care access and digital engagement in the United Kingdom. This approach allowed a comprehensive exploration of common barriers and facilitators affecting both groups. In addition, practical considerations related to recruitment and resource availability supported this grouping, facilitating a more efficient and meaningful discussion. In total, 2 online and 2 in-person focus groups were conducted (1 each for South Asian participants and Black African or Caribbean participants). We anticipated that the online groups may include participants who were more digitally engaged, whereas the in-person groups may include participants who were less engaged or excluded.

Focus groups were facilitated by 2 researchers; the 2 lead focus group researchers came from the same ethnic group as the target populations (NA and BP, supported by AH and JM). Focus groups lasted up to 2 hours (including breaks). Online focus groups were held via Zoom (Zoom Video Communications). In-person groups were held on premises used by the community group through which they were recruited. For the in-person focus groups, we offered participants interpreter support via a gatekeeper from within that community group, but this was not necessary as all participants demonstrated strong proficiency in English. Community groups were reimbursed for the costs of arranging and hosting the focus groups on their premises. The participants received a £50 (US $65) Amazon shopping voucher as compensation for their involvement in the focus group session. This amount was chosen to adhere to the National Institute for Health and Care Research guidelines for payment to participants, ensuring it was appropriate and avoiding any concerns regarding financial coercion [41].

Data were collected in April and May 2023.

### Data Analysis

In total, 4 focus group sessions were recorded on Zoom (online groups) or on an encrypted digital recording device (in-person groups). Data were analyzed using a framework analysis approach. The framework approach was selected for its systematic and flexible nature, which is particularly suited to applied qualitative research that requires findings to be fed back into practice promptly. It allows for both inductive and deductive analysis, accommodating preexisting frameworks while remaining open to new themes emerging from the data [42].

We followed a seven-step framework analysis process [42], as outlined in Textbox 1.

**Seven-step framework analysis process.**

1. Transcription: focus group recordings were professionally transcribed verbatim from high-quality audio, ensuring large margins and spacing for notes.

2. Familiarization: we immersed ourselves in the data through repeated readings to gain a comprehensive understanding of the content. Research team members (NA and AH) read the transcripts for familiarity and rendered them anonymous.

3. Coding: we systematically applied codes to identify relevant features or concepts. Two researchers (NA and AH) coded the same transcript using a broadly inductive approach, staying close to the data.

4. Developing an analytical framework: we grouped the codes into broad thematic categories that aligned with the research questions. Two researchers (NA and AH) agreed upon a coding framework.

5. Applying the framework: we indexed transcripts using agreed codes and categories. Two researchers (NA and JA) applied the framework to the remaining transcripts.

6. Charting data: we organized coded data into a matrix, summarizing it by category. Two researchers (NA and JA) charted the data into a framework matrix using Microsoft Excel.

7. Interpreting data: we explored connections between themes, discussed findings, and derived overarching conclusions. Three researchers (NA, AH, and JA) interpreted the data to identify higher-order themes and similarities and differences.

The stages were iterative, allowing for continuous refinement and revision as the analysis progressed. This structured approach ensured a rigorous, transparent, and comprehensive analysis of the qualitative data, facilitating the identification of meaningful patterns related to the research questions while staying grounded in participants’ experiences.

### Trustworthiness and Rigor

To ensure the rigor and trustworthiness of our qualitative study [43-45], we used several strategies based on the criteria by Lincoln and Guba [44]:

- Credibility—we engaged in prolonged participant interaction to build rapport and gain a comprehensive understanding of their experiences. Several data sources, including focus groups, and varied analytical methods, such as the framework method, were used to cross-verify findings.
- Transferability—rich, thick descriptions of the study context, participants, and findings were provided to enable readers to assess the applicability of our results to other settings.
- Dependability—an audit trail documented all research activities and decisions to facilitate replication and external verification.
- Confirmability—reflexivity was emphasized through ongoing reflection on potential biases, documented in a reflexive journal. Regular peer debriefing sessions refined interpretations and ensured diverse perspectives were considered.

To ensure transparency and rigor in our reporting, we adhered to the guidelines of COREQ (Consolidated Criteria for Reporting Qualitative Research), a 32-item checklist (Multimedia Appendix 2), which are specifically tailored for qualitative studies involving interviews and focus groups. This framework supports a clear and comprehensive presentation of study design, data collection, and analysis, enhancing the credibility and reproducibility of our research [46].

These strategies were collectively used to enhance the trustworthiness and integrity of the research findings.

### Ethical Considerations

Ethics approval was granted by the University of Manchester Proportionate Research Ethics Committee (January 27, 2023; 2023-15589-26902). The research involved human subjects and adhered to institutional policies regarding ethical oversight. All participants provided informed consent before participation. They were informed about the study’s purpose, procedures, and their right to withdraw at any time without their care or legal rights being affected. To protect participants' privacy, all data were fully anonymized. Research team members (NA and AH) reviewed the transcripts and removed any identifying information. Participants received a £50 (US $65) Amazon shopping voucher as a token of appreciation for their time and contributions to the study.

### Patient and Public Involvement and Engagement

In addition to the participatory approach to recruitment, from the onset of this project, the research team engaged with the GMOPN equalities lead and other key members of the network who advised on how the research team might best recruit and engage participants from these communities. Feedback was sought from members of the GMOPN on the recruitment advertisements, interview topic guide, and questions (Multimedia Appendix 1); in addition, we consulted with a project lead and health coordinator from a South Asian community group outside Greater Manchester (Firvale Community Hub, United Kingdom) for advice on the practicalities of conducting in-person focus groups within this community.

The CAHN is an established and experienced community organization that regularly facilitates and leads on recruitment for research participation projects; therefore, it was selected as an expert partner in the project to ensure that we were able to recruit from a diverse pool of older people from Black African and Caribbean descent communities.

## Results

### Participant Characteristics

A total of 27 individuals participated in 4 focus groups (April and May 2023) either in person or via online videoconferencing. Participant characteristics and distribution across focus groups are shown in Table 1. In our study, participants were predominantly older adults, with a median age of 69 years (IQR: 66.5–72.5), with a total of 27 participants, of which 22 (81%) were female and 5 (19%) were male. The South Asian cohort was composed exclusively of individuals of Gujarati Indian descent. This contrasts with the findings of Small et al [47], whose participants primarily represented Pakistani or Bangladeshi heritage. These demographic characteristics highlight the unique perspectives and experiences shared by our participants in the context of digital health access and the impacts of the COVID-19 pandemic.

**Table 1:** Participant characteristics (N=27).

Demographic category		Value
Age (y), median (IQR)		69 (66.5-72.5)
**Sex, n (%)**		
	Female	22 (81)
	Male	5 (19)
**Ethnicity, n (%)**		
	Asian or Asian British Indian	11 (41)
	Asian or Asian British Pakistani	4 (15)
	Black, African, Caribbean, or Black British African	6 (22)
	Black, African, Caribbean, or Black British Caribbean	5 (19)
	Any other Black, African, or Caribbean background (not described)	1 (4)
**Focus group distribution, n (%)**		
	In person: South Asian	10 (37)
	In person: Black African or Caribbean	8 (30)
	Online: South Asian	5 (19)
	Online: Black African or Caribbean	4 (15)

### Qualitative Themes

#### Overview

Findings are organized around 4 prominent themes that we developed inductively from the data (Textbox 2): (1) service accessibility through digital health, (2) importance of face-to-face (in-person) appointments for patient-clinician interactions, (3) stressors caused by the shift to remote access, and (4) digital solutions (evaluating technology acceptance).

**Overview of Key Themes in Digital Health Access and Implications for Older Adults in the United Kingdom (April-May 2023)**

This chart presents the 4 main themes identified from focus group discussions on digital health services among older adults (aged >65 years) from South Asian, Black African, and Caribbean backgrounds in the United Kingdom. The study was conducted from April to May 2023, involving both web-based and in-person focus groups.

- **Theme 1: Service accessibility through digital health**
  Challenges faced by participants, including language barriers and limited digital skills, reliance on younger family members or community organizations for assistance, and concerns about digital health services being primarily tailored for English speakers with insufficient consultation during development.
- **Theme 2: Importance of face-to-face (in-person) appointments**
  The high value placed on in-person interactions with health care providers. Although video consultations were considered acceptable alternative, face-to-face in-person meetings were strongly preferred.
- **Theme 3: Stressors caused by the shift to remote access**
  Participants experienced stress, fear, and anxiety due to the shift to remote digital health services, feeling that these solutions were imposed without sufficient explanation or consent. Additionally, Black African and Caribbean participants reported facing racial discrimination within the health care system.
- **Theme 4: Digital solutions: evaluating technology acceptance**
  Acknowledgment of the benefits of digital health services, coupled with caution against a one-size-fits-all approach. Participants advocated for offline alternatives and a hybrid model, highlighting the need for choice and adequate support for the clinical workforce.

**Theme 1: Service accessibility through digital health**

- Accessibility extent
  - Procedural issues
    - Booking appointments
  - Language and cultural issues
  - Digital skills issues
  - Access to services
    General practices
    Dentists
    Pharmacies
    Other community services

**Theme 2: Importance of face-to-face (in-person) appointments**

- Importance of in-person interactions
  - Value of face-to-face meetings
    - GP's ability to see patients
  - Skepticism of telephone appointments
  - Emphasis on patient-clinician interaction

**Theme 3: Stressors caused by the shift to remote access**

- Psychosocial impacts
  - Influence on thoughts, emotions, and behaviors
    Increased stress
    Agitation
    Frustration
    Anxiety
  - Social Interactions and environment
  - Impact on mental well-being
    Concerns about technology use
      Difficulty in making health appointments

**Theme 4: Digital solutions: evaluating technology acceptance**

- Extent of technology acceptance
  Digital access and delivery of health services
    Viewed as appropriate
    Digitalization seen as the future
    Caution against one-size-fits-all
  Acknowledgment of diverse needs
    Advocacy for offline alternatives
  Concerns about sensitive issues
    Preference for in-person discussion
  Importance of choice
    Hybrid approach suggested
      Options for mode of consultation

In the quotations provided, participants are identified by their unique identifier (eg, P1=participant 1 or P24=participant 24), their ethnicity (eg, SA=South Asian or BAC=Black African and Caribbean), and the mode of focus group in which they took part (eg, I=in-person focus group and O=online focus group).

#### Theme 1: Service Accessibility Through Digital Health

##### Overview

This prominent theme covers the extent to which participants felt that digital and remote access to health services is accessible (eg, through a phone call or via online platforms). It covers procedural issues about booking appointments; issues relating to language and culture; issues relating to digital skills; and access to dentists, pharmacies, and other community services. These issues emerged consistently across all 4 focus group sessions.

##### Appointment Booking

Many participants referred to trying (and often failing) to telephone GPs at 8 AM to get an appointment, leading to more people going to accident and emergency departments. This issue was perceived to have worsened during the COVID-19 pandemic:

What’s happening is people who cannot access their GPs end up going to A&E, which is costing a lot more, which is impacting on the ambulances not getting out to people and not being able to deal with people who have got real emergencies...it’s a broken system.

P23, SA/O

Both online and in-person groups reported challenges in accessing GP services, with difficulties in booking appointments and navigating gatekeeping by receptionists. Some participants felt that the role of the receptionist has become more of a gatekeeping role, in which receptionists ask what are perceived to be sometimes inappropriate, personal, or sensitive questions about symptoms and reasons for wanting a GP appointment. South Asian participants were concerned about discussing sensitive health issues, illustrated by a discussion mainly in the in-person focus group:

They ask you.

P1, SA/I

That’s right.

P5, SA/I

They say what is the symptoms...

P6, SA/I

...That’s true, sometimes it’s private and confidential things...now reception wants to know before you see the doctor.

P8, SA/I

Yes, that’s right. Yeah.

P5, SA/I

...Sometimes we have to say if we don’t want to tell them...I just say it’s my personal...then she would say...what is the symptoms.

P9, SA/I

There were also concerns about the flow of communication from the receptionist to the physician; delayed appointments; and lack of continuity of care, that is, not being able to see the same physician, particularly after hospital discharge. These concerns emerged consistently across all 4 focus groups but predominantly among the South Asian participants (both in-person and online groups). Some South Asian participants highlighted how booking appointments via online platforms may be beneficial if it ensured rapid triage and bypassed receptionists who are perceived to be intrusive or uncaring.

Participants raised some concerns about older people being unable to access dental services, which was reported as having become more prevalent during the COVID-19 pandemic:

Some of them are really, really suffering with the pain, especially if they’ve got infections or gum problems. And they couldn’t go because of the COVID...now they are trying to go private, aren’t they...unless you have been registered and if you’ve not been using the service, then they take your name off, right...And there is a lot of elderly are going through...especially when they have loose tooth, they can’t afford it, they want to pay privately, there’s so many issues with that.

P4, SA/I

Some participants were unsure how to make an online dental appointment or whom to contact for assistance, leading to a feeling that they had no choice but to tolerate unresolved dental issues.

##### Language and Culture

There was a strong sense that language difficulties made remote access to health services difficult for older people who do not speak English as a first language, both in terms of attempting to use online platforms to access appointments or health information and also if they needed to explain medical symptoms remotely. Participants in both online and in-person groups reported difficulties in accessing digital health care services, with a notable concern for language barriers and digital literacy:

And what about people who are not English? And when I say not English, like Jamaican, African, not...the English language, it’s second for them. There’re loads of problems really that nobody’s bothering about. What about uneducated people?

P27, BAC/O

Both online and in-person groups faced challenges with language barriers. Some participants suggested that it might be useful if information and interactive services were available in different languages. However, others were mindful that low first-language literacy among some older people in their communities meant that this would not suit everybody and pointed out the different levels of literacy within and across the different communities:

...that South Asian community which came here in 1950s onwards, the majority are from small towns and villages and now they are as a first generation—so literacy is only 23 percent. Most of them cannot even read and write their own language so how they can use...and how many people have a smart phone or internet access?

P20, SA/O

The role of interpreters was discussed and highlighted as not straightforward, as families cannot interpret for relatives because of conflict of interest, family pressures, impartiality, and confidentiality issues:

...at the moment, I think this is a new thing that family cannot interpret because I have to go with my mum many times and because I live with her, I’m official carer, but family can put pressure and it has to be the interest of the person. So, I cannot interpret. They have to get an outside interpreter.

P7, SA/I

There was an overall belief across all 4 focus groups that some older people were missing out on access to essential services and health care provision because of language barriers, which compound digital exclusion. Participants from all 3 ethnic groups highlighted the need for culturally sensitive digital health services. There was a perception that online systems and technology-facilitated access have been designed for predominantly “White people” or those who are proficient in English, with concerns expressed about a lack of consultation during the development stages:

Now, when NHS or these health ministers, if they are giving the contract to somebody to design the app for this kind of thing, they must give them first and make sure they have a cultural competence training before they design anything. They should know whom they’re designing for. They are not just designing for white people, those who can speak English or Asians who can speak English, it is for everybody.

P20, SA/O

Participants here also raised concerns about “rolling out” of technology and the lack of public awareness and access to the various options available.

##### Digital Skills and Training

Participants talked extensively about (a lack of) digital skills and the type of training that might be beneficial, with online groups discussing the benefits of digital champions, while in-person groups mentioned reliance on younger family members for support. Many participants reported that they and other older people in their communities often needed support from friends and family to engage in online interactions with health services. Some participants received support from community groups to access online health services as part of broader digital skills training programs, which included using Zoom for exercise sessions, healthy living initiatives, and family communication. However, this was not always straightforward, as some participants struggled with online medication orders and repeat prescriptions. For other participants, using the telephone instead of online services was also challenging because of hearing difficulties.

Some participants felt the NHS app has been helpful because patients do not have to wait for telephone calls about appointments or test results and can receive reminders, which they find helpful:

...we have got the NHS app so any messages they send me online and I can order my repeat prescription...It’s like when you have a blood test, when I phoned the next day, the results go on the app...If you’ve got an appointment, they send you on your phone and a letter and then it reminds us two days before. I find this helpful.

P12, BAC/I

However, there were other examples of people being unaware that test results were communicated via the app and thought that they were left waiting. There were also concerns about how results would be interpreted on apps, compounded by an unfamiliarity with medical terms:

...some of us don’t even know we go there and then you have your bloods done and then you’re sat at home waiting for the results, and the next time you go they say, what happened to my tests, they say, oh, it’s on the app. I said which app?

P17, BAC/I

Others indicated that the NHS app use has proved problematic, either because of issues with downloading or with password issues, for example, forgotten passwords and a lack of knowledge about how to request a new one.

Participants were positive about the role of local digital champion schemes and endorsed the idea of befrienders, buddy systems, volunteer, and patient participation groups to help deliver training sessions to older people. Overall, the role of (usually younger) relatives and members of community organizations to support people digitally (both in general and around health appointments or prescriptions) was cited as being important. However, it is not clear how appropriate this might be in the context of health care in upholding privacy:

...if she has got any gynae issue it is very hard for that Asian mother to ask her son to put that something into this app. Because it’s...like cultural wise it’s kind of a private things when it comes to gynaecological or sexual issues. Parents do not talk. Maybe mother is easy to talk with the daughter but not with the son, it’s out of the question. So, these things they have to consider.

P20, SA/O

This example referred to women making gynecological appointments, but it is equally plausible that older men may have privacy needs.

#### Theme 2: Importance of Face-to-Face (In-Person) Appointments for Patient-Clinician Interactions

This theme highlights the extent to which participants felt that having an in-person, face-to-face interaction with their clinicians (particularly a GP) is important. There were multiple quotes emphasizing the importance of the GP being able to see patients and skepticism of telephone appointments:

...and if they are doing some face-to-face, the relationship with the doctor has been real, they will be able to recognise that very quickly. And at least identify the problem much quicker than being on the phone.

P3, SA/I

There was a strong preference for in-person appointments among all focus groups, as participants queried how well GPs might understand a problem if they cannot see the patient. In-person, face-to-face appointments were strongly preferred for the value of physical interaction and connection (direct patient-clinician interaction), which was felt to be less tangible in online settings:

...how can they diagnose something when they don’t see you, my husband can’t hear it properly... ‘cause we can’t pronounce things...like to see face-to-face that we can say it. How to speak...I don’t know how to spell everything.

P2, SA/I

The other advantage of face-to-face, somebody could be suicidal, depressed, you talk to somebody over the phone, to the doctor, they can’t see your face.

P12, BAC/I

Others suggested that a face-to-face consultation with their GPs was important at an interpersonal level and was considered “uplifting” and “healing.” This was also related to some of the perspectives about language challenges and the feeling that some older people may be able to explain problems much better in person rather than through online platforms or over the telephone:

We are not [confident], so we want that line that will connect with our GPs, because really to be honest, when you go to your GP, you see that GP face-to-face, you feel already better, whereas when you’re online, there’s no connection, there’s no relationship for us. It’s just machine noise.

P18, BAC/I

Although there was limited experience of video consultations with GPs, some participants were positive about this mode of consultation and perceived it to be a viable alternative, the next best thing to an in-person appointment:

...well for a start, I think there’s nothing better than having face-to-face communication, right. But talking about FaceTime or video call, we’ve had this experience where my father-in-law fell, and when the sister-in-law rang the doctor, obviously the doctor came on video call, it was over...due to COVID, and because she was on a video call, the doctor wanted to see exactly what happened, and the doctor was able to diagnose to say that he’s actually fractured his hip, because she could actually see exactly what was happening. And she called the ambulance and everything. And he was taken to hospital. But if you can’t contact or have communication with doctor, face-to-face, at least having video call would probably be another best thing.

P5, SA/I

However, the following quote highlights the importance and usefulness of an in-person face-to-face approach for “seeing and feeling” patients; this seems to be the preferred method for undertaking regular patient reviews and for monitoring purposes:

...you cannot have regular reviews online because they need to see you, they need to monitor you, you need to take those tests, they need to see your grip, they need to see your stand, they need to see your position...they need to smell your breath, you know what I mean? Just need to know what you’re like. Because sometimes people’s breath smells of pear drops and that’s a sign of diabetes or some kind of ailment I think. But they need to be there. And if it smells of almonds you know...there’s something wrong with them and whatever.

P24, BAC/O

Participants also acknowledged advantages of using video technology for some people (eg, if they live far away), as many older people may have transportation issues and may find in-person appointments challenging:

But this is what would be useful, if the GPs use the FaceTime. If they use the FaceTime with the patients...the patient doesn’t have to worry about transport, which the majority...of them have the problem. If the GP...they don’t even really need the receptionist, they just say, right...this is the doctor, I want to see you, can you give me time. That’s all they have to say. And if the FaceTime...it can be...not hundred per cent but near enough to face-to-face conversation. So that would be a very big asset.

P10, SA/I

Video consultation was seen as a familiar mode of communication, as participants were able to relate it to remote interaction with relatives during the COVID-19 pandemic.

#### Theme 3: Stressors Caused by the Shift to Remote Access

This theme captures the often negative psychosocial impacts (encompassing the influence of social interactions and environment on thoughts, emotions, and behaviors, along with their effect on mental well-being) reported by people due to the shift to remote appointments and appointment booking. Participants expressed multiple concerns about using technology to schedule health appointments, noting that it increased their stress, agitation, frustration, and anxiety over their inability to secure appointments:

For me, it’s scary, technology, because it changes so fast, you can’t cope and like they’ve said, the person-to-person thing, you get connected with your doctor and you’ve got answers when you go there, you’ve got answers. But the machine will go on, you keep carrying on half an hour, 40 minutes, you’re next in the line, you’re next in the queue, you’re number three, whatever, you wait and wait and wait, and then you lose patience...so, technology is scary because we get also anxious...I’m scared of the machine. I’m not able to grasp it...it’s so stressful.

P18, BAC/I

These experiences were felt by some to contribute to a deeper fear of becoming ill in the first place:

I think it’s [the current health system] more stressful. Because you feared to become ill. You fear to become ill because doctors and national health doors are closed now. Those who can be...you know, get to their...they...we don’t know the digital, we can’t get any Internet and we can’t call them, then every...all the doors are closed for us, so it’s a fearful life.

P9, SA/I

Several participants commented on how the “old days” were better, where they could walk up to their GP’s office and sit in a queue to be seen by a GP:

In my opinion, yeah, it was better before. During COVID and after COVID it’s gone worse, you know, in my experience, you know. Because when you need an emergency care, you know, you need to see your GP, you need to see the professional, you know, and if you don’t get the chance to see them, you know, it will affect your mental health as well.

P23, SA/O

Some participants felt that digital access to health care has been “thrown at them” and that it will take time for them to adjust to this new way of working:

I think because we were used to the old system of doing things, this one has just been thrown at us, and as we keep saying, it’s scary. It will take time for us to move into there and then it has to be simplified as well for us because if you ask me about getting in touch online to my GP, I personally don’t like it.

P15, BAC/I

There were concerns about consent and the “small print,” with the sense that people may be very confused about what is happening with their data when engaging with the health service digitally:

I don’t like to go online because of all the information...actually I don’t trust the online services because things get leaked and so forth online. Personally, why it’s a bit unfair, is that most older people will not read the small print of anything that’s put online. They will just complete the form and they don’t know if they’re signing their life away. And because they haven’t read the small print anything can happen. You know what I mean? And when it comes to your health and the national health, imagine if that small print said if you fill in this form, that you’ve agreed to your organs being donated, when they don’t want to do that.

P24, BAC/O

This sense of fear and confusion of using technologies to access health care was cited within a wider narrative of fear and lack of confidence in using technologies more generally (eg, fear of online banking and scams):

...there’s too many scams going about now. The older people, they are really getting scared of that. And not even the older, the younger people...The scams are so sophisticated to do with computers that I couldn’t imagine what could go wrong if some person had all the national health number, this number, that...

P27, BAC/O

Participants describe their fear of missed opportunities, late diagnosis, and neglect, which they felt were creating more health inequalities for older people because of the digitalization of services:

...people, especially with South Asians, if they can’t access their GPs and they can’t use the app what’s happening is, if they’ve got a condition, they’re going to let it fester until it gets to a point where they have to go into hospital. So, it’s actually going to cost you more and really, you know, there should be other pathways that people...like we’ve said, you know, that they can’t get into the GPs but there should be at least somewhere. Some health professional, you know, triaging this and saying, you know, let’s get you seen by a nurse or see what is the issue? And I don’t think you can just rely on apps and online technology, I think there’s always going to be a percentage of people who are not going to be able to access, and that’s, you know, in a way you’re creating more health inequalities.

P19, SA/O

The topic of institutional racism and discrimination within the health service was raised by participants in the Black African and Caribbean groups, with some feeling that they are not treated with sympathy, empathy, or respect when making appointments. For example, there were descriptions of being treated differently, both over the telephone and in person, because of being identified as Black or “not British”:

We’re living in a very stressful society now and the assumption that it’s easy to access the GP or NHS, it’s not. It’s not...They look at you, institutional racism...They’re not going to deal with you with sympathy, empathy, with respect. I’ve seen it. I’ve seen, I’m not assuming, you know? I know there are challenges because they look at you, she’s old, no disrespect, she’s Black, they don’t know the technology, they don’t understand it...they hear your voice, oh no, this one is not British, okay, I’m going to treat that person differently. And she gets angry at that because to take a phone to make a call to a person you don’t know to express what you’re feeling and what you want, it takes some courage, that has to be respected.

P15, BAC/I

This quote also emphasizes the intimidation people may feel when trying to express their concerns and emotions over the phone to an unfamiliar person, highlighting how this discomfort is often not acknowledged or respected by health care professionals. There were some reflections on cultural gender differences, with examples of digitally excluded Asian men possibly being further excluded because they are more reluctant to seek health care than women. However, these reflections were made with the caveat that the vast majority of study participants were women.

#### Theme 4: Digital Solutions (Evaluating Technology Acceptance)

This theme captures the extent to which participants felt that digital access and delivery of health services are appropriate. Participants accept that digitalization is the way forward but caution against regarding it as a one-size-fits-all solution, acknowledging the diversity of individual needs and advocating for offline alternatives.

Relating back to the theme about accessibility, there were some concerns that more personal or private health issues (eg, sexual or reproductive health and mental health) would be better suited to in-person discussion. Choice was seen as very important, and participants felt that people should be given options about mode of consultation, with some suggesting adopting a “hybrid approach” as a way forward:

I think we should be given options. Do you want to come face-to-face? Do you want telephone consultation? Do you want this? We are different, we all have different strengths and weaknesses.

P15, BAC/I

I think there should be a hybrid approach, you know, there should be options for those people who can’t access technology. There should be somewhere that they are either supported in the GP surgery to be able to do that. And whether that’s...I don’t know, I don’t want to go back to community pharmacies because they’re already busy doing everything else. So, whether...those people that can do it online or through other ways...

P19, SA/O

Some participants were skeptical about the potential benefits of technology-based access to the health service if there was a fundamental lack of a clinical workforce to deliver care:

I think the technology was to help improve the system, but the problem has been you’ve got a reduced workforce, so you haven’t got the staff there to actually deal with all these...online technology and the requests coming through. I don’t think it’s just been from COVID; I think it’s been over the last 10 or 12 years it’s been reduced, the staff and the workforce...

P23, SA/O

These quotes show that participants recognized workforce challenges as a longer-term issue that predated the COVID-19 pandemic and that affected all members of society, not just specific ethnic groups.

## Discussion

### Principal Findings

This study specifically focused on the experiences and views of older adults from South Asian, Black African, and Caribbean backgrounds regarding the digitalization of primary care services since the COVID-19 pandemic. While previous research has explored digital health inequalities [47], this study specifically addressed the gap in qualitative evidence from the United Kingdom by concentrating on these older adults’ perceptions and experiences with digital health since the COVID-19 pandemic began. It underscores the need for a detailed examination of the impacts of COVID-19 pandemic–driven digitalization on health services and potential health inequalities among older people from these minoritized ethnic groups. The study aimed to understand how digital access to health and care services was experienced by diverse groups of older people to tackle digital exclusion through targeted interventions, cultural considerations, and ongoing support for equitable digital access and engagement. While our study specifically targets older individuals (as opposed to a broader adult demographic), the results closely parallel those of Small et al [47], which examined digital health experiences among older adults primarily of Pakistani and Bangladeshi heritage. Their findings highlighted significant barriers faced by these groups in accessing digital health care services, similar to our participants’ experiences with language barriers and the preference for in-person consultations. These similarities underscore the pervasive nature of digital health inequalities across different minoritized ethnic groups, emphasizing the urgent need for culturally sensitive approaches to digital health care access. It is noteworthy that most of their participants were of Pakistani or Bangladeshi heritage, whereas our study’s South Asian cohort exclusively comprised individuals of Guajarati Indian descent.

The finding that many participants reported trying and failing repeatedly during, and since, the COVID-19 pandemic to get through to their GP to make an appointment is unsurprising. It is widely acknowledged by both the UK public and policy makers that ending the “8 AM rush” of phone calls in the morning is a key objective of the recent NHS England guidelines aimed at improving access to primary care in the postpandemic recovery phase. [48]. This guidance proposes implementing “modern general practice access” via digital telephony and online requests as a key approach to realizing this ambition. Although this may be helpful for many people, our study reveals strong concerns about language barriers for older people who do not speak English as a first language, both in attempting to use online platforms to access appointments or information and in using the telephone to book appointments or speak with clinicians. This finding reinforces work conducted before the COVID-19 pandemic, showing the communicative disadvantages that digital or remote consultation tools and access to health services can place upon certain people [28,49]. In our study, the role of (usually younger) relatives and members of community organizations to support older people to overcome digital and language barriers around health appointments was highlighted as being important, but a reliance on this support may be inappropriate in some contexts that are deemed to be more personal, for example, women seeking gynecological appointments. It is also not clear that simply offering digital services in different languages will be helpful for everyone, as participants in our study cautioned that literacy levels among some older members of their communities are low.

The role of receptionists as gatekeepers has been a longstanding issue; for example, research conducted >20 years ago identified challenges faced by receptionists in managing patient appointments and revealed discriminatory behavior toward certain patient groups [50]. Some participants felt that the role of the GP receptionist has changed over the COVID-19 pandemic to become more that of a gatekeeper, and they queried the appropriateness of some of the questions they had been asked about why they wanted an appointment. Black African and Caribbean participants specifically reported experiencing discrimination that impacted their access to health care. Some of the Black African and Caribbean participants raised concerns about racism and discrimination in the health service during both face-to-face and remote access, which have been reported in other qualitative work by our research group exploring access to palliative and end-of-life care during the COVID-19 pandemic for people of Black African and Caribbean backgrounds [50]. It is concerning that some participants in our study reported that these issues are still occurring.

Several participants emphasized the importance of visual interaction, whether through video meetings or in-person meetings (face-face), with clinicians. They expressed a strong preference for in-person appointments, which was linked to concerns about language barriers and the ability to explain problems over the telephone. This finding supports early COVID-19 pandemic longitudinal research, which showed that in some practices with large older, immigrant populations considered deprived, remote telephone consultations were more challenging than face-to-face consultations due to the importance of nonverbal cues [51]. It also echoes work with older patients with musculoskeletal conditions who expressed dissatisfaction with remote consultations for new diagnoses or in-depth discussions, emphasizing the necessity and importance of face-to-face interactions in developing the therapeutic relationship [52]. Participants in our study were positive about video consultations as the next best thing to in-person consultations because of the ability of this technology to facilitate visual contact with clinicians. Work conducted on video consultations before the COVID-19 pandemic has largely involved patients considered low risk managing stable long-term conditions, with limited relevance to wide-scale changes in general practice service delivery [11]. Our findings reinforce work conducted toward the beginning of the COVID-19 pandemic that highlights that there may be times when visual interaction, either by video meeting or in-person face-to-face interaction with clinicians, may be felt to be more appropriate [51-53]. Practices with greater numbers of older patients appear to perceive a lower digital confidence in their patients, which may negatively influence the digital readiness of practice staff [54]. Therefore, it is important to explore further the situations in which video consultation is felt to have a clear advantage to either telephone or in-person consultation, which may include patient preference. Some challenges that relate to communication and rapport in remote consultations are more general to an aging population, for example, for people with hearing loss, and others are specific to minoritized ethnic groups, for example, for people with language difficulties. However, general challenges may be exacerbated by specific challenges; for example, an older adult from a minoritized ethnic group who has hearing loss may experience further difficulties in hearing in a language that is not their first language. Video consultations may provide a window for some visual cues, but their usefulness for some patient groups, for example, dementia services, has been questioned as they do not offer the same sensory experience as face-to-face consultations [53]. In the absence of other sensory data (eg, physical examination), remote consultations rely more on the patient’s ability to report their history, and hence, patients who struggle to communicate in the manner and language expected by the clinician can be particularly vulnerable [27]. Findings from our study suggested that older people from minoritized ethnic groups may fall into this vulnerable category. Language use varies significantly across contexts, and understanding these variations, such as the formal consultative register often used in professional settings, can help elucidate the communication challenges faced by older adults, particularly in health care interactions. This is essential, as effective communication is key to ensuring that patients feel understood and supported in their health care experiences [55]. Some participants reported negative impacts of struggling to get appointments, including stress, frustration, and a deeper fear of becoming ill in the first place. Health care policy and research have long focused on mitigating missed appointments, primarily through the lens of improving resource efficiency. A recent evidence review of nonattendance in the NHS shows a clear age, socioeconomic, and health inequalities gradient, as older people from lower socioeconomic groups with multiple long-term conditions are most likely to miss appointments [56]. The review suggests that addressing inefficiencies in the appointment booking system and enhanced patient involvement in the booking process may help address the problem. Our study suggests that the impacts on older people’s well-being of being unable to arrange appointments in the first place may need further investigation. The recent “major conditions strategy” emphasizes early diagnosis, early intervention, and quality treatment [57]. Our findings raise concerns that individuals may struggle to schedule appointments and receive timely GP treatment, leading to increased reliance on hospital resources, either in accident and emergency departments or in the long term when untreated conditions worsen.

Participants generally agreed that digital solutions are the way forward and recognized the benefits of digital access for many individuals, such as time savings and convenience. However, they cautioned against perceiving technology as a universal solution and advocated for offline alternatives with the option to access health care in different ways. The importance of support systems at the practice level to assist individuals struggling or encountering difficulties with digital access was a recurring theme among South Asian, Black African, and Caribbean communities.

When comparing the responses of participants in online and in-person focus groups, several similarities and differences can be identified. South Asian and Black African and Caribbean participants express common challenges in navigating digital health platforms and accessing primary health care services. Issues such as technological barriers and disparities in digital literacy underscore the need for tailored approaches to enhance accessibility and usability for diverse populations. Participants’ opinions and experiences of digital health and primary health care access are shaped by many cultural influences. South Asian participants frequently emphasize the relevance of familial and community networks in health care decision-making, whereas Black African and Caribbean participants highlight historical circumstances such as migration histories and prejudice, including discrimination. These cultural differences influence communication methods and health care seeking behaviors as well as engagement with digital health services.

When comparing online and in-person contact with health professionals, distinct factors come into play. In online interactions, the lack of nonverbal cues might impact communication dynamics, potentially presenting difficulties, especially for South Asian participants who often rely on subtle cues. Conversely, in-person consultations encourage spontaneous exchanges, but practical barriers such as the need for physical attendance may limit participation to those who can travel to the location, affecting specific demographic groups.

Understanding these nuances is essential for fostering digital health equity and increasing primary care access. To achieve inclusive and effective digital health solutions, researchers and health care professionals must consider cultural preferences as well as technological barriers and constraints. Tailored interventions, informed by insights from diverse communities, can enhance engagement and empower individuals to make informed health care decisions.

Our work adds to the literature emphasizing the need for ongoing support from the community and adopting a hybrid approach that considers both in-person and online or digital interactions aiming to address exclusion while preserving face-to-face options [12-16,19,25,58-62]. Therefore, it is encouraging to see that this is reflected in the NHS primary care recovery plan’s emphasis that patients will “always have the option of visiting their practice in person” [48]. The plan also emphasizes increasing knowledge and confidence in the use of the NHS app and other digital access routes. Participants in our study called for more support to increase digital skills and confidence among older people and indicated willingness to learn. Other work has reinforced these calls and has advocated for culturally appropriate community support [13,58]. However, it is also important to consider the design of technologies, long before the point of training and support; participants in our study raised concerns about the extent to which they felt digitalized services have been designed in consultation with older people from minoritized ethnic groups and for whom English is not a first language. The design of digital health technologies should be informed by user experience (UX) design methodology, which aims to understand the needs, behaviors, motivations, and preferences of users [63,64], but in the development of digital health care, UX is frequently underestimated [64]. By applying these principles, developers can create digital health services that are more intuitive and accessible, particularly for older adults from minoritized ethnic groups who may face additional barriers to engagement. A user-centered approach is vital for addressing challenges such as language barriers and varying digital literacy levels. By integrating UX design principles, digital health solutions can achieve better health outcomes and greater equity. Despite its importance, UX is often underestimated in digital health care development. Effective user research is crucial for minimizing risks, enhancing satisfaction, and ensuring successful product adoption [65]. Participants urged policy makers to consider this more carefully. Further research is needed to explore strategies for enhancing digital health literacy and promoting equitable access to primary health care services. By prioritizing inclusivity and cultural competence, health care systems can better meet the diverse needs of South Asian and Black African and Caribbean populations, ultimately advancing health equity and improving health outcomes.

### Implications and Recommendations

This study offers insights into the experiences of older adults at higher risk of digital exclusion, emphasizing the need for inclusivity in digital health initiatives. Future research should focus on the perspectives of digitally excluded individuals for more effective interventions. Several suggestions arise from our work for policy makers to consider regarding the future of digitalized health and care services. The main recommendation is that the design of digital health services must involve in-depth consultation and UX research with older adults from diverse backgrounds, including those with limited English language proficiency. It is important to not underestimate the impact of language barriers and the challenges this can pose for digital literacy and engagement, and simply offering translation functions within digital technologies may be insufficient. It is also important to recognize that although older adults may be able to ask friends or family for support in using digital technologies and may be willing to learn how to use digital technologies, there are times when this will not be appropriate as people need privacy to raise intimate health issues. More simple queries may be addressed through online platforms or via telephone, but face-to-face or visual contact with clinicians is important to allow patients to feel confident that their problems are understood and to develop a therapeutic relationship. This may be particularly important for older people who have limited or no English language skills and particularly important for certain health issues. Regarding training and support, the use of buddies or champions at a practice level may warrant further consideration and evaluation to help support people who are having difficulty accessing services.

### Strengths

Ensuring data trustworthiness in qualitative research, especially in focus group studies, is crucial for valid findings. Thematic analysis, advocated by Nowell et al [43], helps identify patterns and themes in participants’ narratives. Lincoln and Guba [44] stress 4 key criteria: credibility, transferability, dependability, and confirmability. Ahmed [45] adds transparency, reflexivity, and ethics. The framework analysis method applied in this study adheres to these criteria [42]. It enhances credibility through structured analysis, promotes transferability via transparent documentation, ensures dependability and confirmability through consistency and systematic checks, and encourages reflexivity and ethics. Overall, adhering to these principles strengthens research integrity and advances knowledge in the field.

Using a participatory approach in this study is a strength as it fosters greater engagement from participants, leading to more meaningful data and insights. This collaborative approach enhances the validity of the research findings by incorporating diverse perspectives and broadening the range of perspectives represented in our study population. We used various methods, including collaboration with community organizations, leveraging an online recruitment platform (Valuing Our Intellectual Capital and Experience Voice), and tapping into personal networks. By partnering closely with community organizations, we elevated their role in our research, allowing them to shape our study and comprehend its outcomes. This collaborative approach extends beyond typical researcher-participant dynamics, fostering a sense of ownership among all involved. Involving community organizations enables us to amplify voices often overlooked in research, ensuring inclusivity and fairness. Rather than simply gathering data, we engage in ongoing dialogues and partnerships to deeply understand diverse perspectives, reflecting our commitment to values such as fairness, equality, and respect for all viewpoints, ultimately enhancing the relevance and impact of our research across diverse communities. This meticulous approach not only facilitated participant engagement but also broadened the spectrum of perspectives, enriching the depth and breadth of our data.

Despite comprising only a small portion of our total participants, the inclusion of individuals aged >75 years was purposeful, aiming to gain valuable insights into the experiences of older adults, an often-overlooked demographic [66]. Their involvement ensures the relevance of our findings to this age group.

We selected focus groups for their ability to provide diverse perspectives and stimulate rich discussions through group dynamics. They offer cost-effectiveness and efficiency in data collection. While they give a broad overview, in-depth insights are still achievable with a skilled facilitator encouraging detailed discussions.

### Limitations

Despite this participatory approach, the main limitation of the study is the extent to which we reached digitally excluded older adults. We aimed to recruit participants from across the spectrum of digital engagement, but the qualitative data suggest that the levels of engagement appeared to be similar across the in-person and online groups. While this study represents a crucial step toward understanding the digital health experiences of 3 minoritized ethnic groups, we acknowledge the need for further exploration, particularly in delving deeper into the experiences of digitally excluded individuals.

We had also hoped to recruit people who may not have been proficient in use of English, given the impact that language has on digital exclusion, and we did not include any quantitative measure of participants’ levels of digital health literacy (eg, via the eHealth Literacy Scale) [67,68] because it has not been translated into appropriate languages. However, all participants had a good level of English, and therefore, it is likely that we did not reach people who may be the most digitally excluded. Despite this limitation, participants offered some valuable insights into the dual nature of digital and language exclusion when attempting to access health services via digital or remote means. Finally, the vast majority (22/27, 81%) of participants were women, so we are unable to say much about gender-based norms and expectations within each ethnic group.

### Conclusions

The COVID-19 pandemic from 2020 to 2022 led governments worldwide to impose lockdowns and social restrictions while also rapidly introducing digital health and care services. However, concerns emerge regarding the possible marginalization of older adults, who encounter significant obstacles to digital inclusion, such as age, socioeconomic status, literacy levels, and ethnicity. The study concludes that digital health initiatives should address the digital divide and health inequalities, ensuring accessibility, choice, and privacy for older adults from these backgrounds. This study highlights the importance of designing digital primary health services through extensive consultation with older adults from diverse backgrounds. Crucially, addressing language barriers requires more than merely offering translation functions. The assumption that younger relatives or members of communities will be able to support older people to engage digitally can be misplaced, and maintaining options for in-person consultations, particularly for intimate or sensitive health care issues, is crucial. There is a need for a flexible approach that combines both traditional and digital health care choices, rather than a one-size-fits-all digital strategy.

## Appendix

Multimedia Appendix 1 Topic guides for in-person and online groups.

Multimedia Appendix 2 COREQ (Consolidated Criteria for Reporting Qualitative Research) guidelines.

## Abbreviations

CAHN: Caribbean and African Health Network
COREQ: Consolidated Criteria for Reporting Qualitative Research
DHI: digital health information
GMOPN: Greater Manchester Older People’s Network
GP: general practitioner
NHS: National Health Service
UX: user experience
WHO: World Health Organization

## Data Availability

The datasets generated and analyzed in this study are not publicly available due to confidentiality concerns regarding participant information. However, they can be obtained from the corresponding author upon reasonable request.
