# Supplementary material for: Experiences and Views of Older Adults of South Asian, Black African, and Caribbean Backgrounds About the Digitalization of Primary Care Services Since the COVID-19 Pandemic: Qualitative Focus Group Study
Source: JMIR Form Res. 2024 Dec 18;8:e57580. doi: 10.2196/57580 (PMC11656993; doi:10.2196/57580)
Supplement: Multimedia Appendix 1 [file formative_v8i1e57580_app1.docx]

| 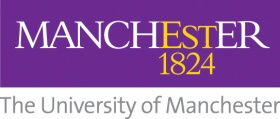 | 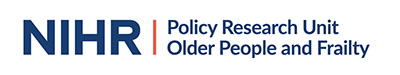 |
| --- | --- |

**In Person Focus Group Topic Guide**

**Using Digital Health Technologies to Access Primary Care**

**In Person Focus Group Topic Guides**

*Indicative topic guide: questions may not necessarily be asked verbatim or in the specific order they appear*

**---------------------------------------------------------------------------------------------------------------**

**INTRODUCTION**

**Welcome**

- Thank group for helping out today.
- Introduce research team and department.
- *This study is being conducted by the National Institute for Health and Care Research (NIHR) Older People and Frailty Policy Research Unit on behalf of the Department of Health and Social Care.*
- *The NIHR Older People and Frailty Policy Research Unit works to produce timely, high-quality evidence for policymakers.*
- *We are a collaboration between the University of Manchester, Newcastle University, and the London School of Economics.*
- *We answer policymakers’ questions and help the Department of Health and Social Care make informed decisions about the lives of older people.*
- *We carry out research into promoting healthy ageing, the future needs of older people and the provision of high-quality, cost-effective care.*
- *The Older People and Frailty Policy Research Unity is funded by the NIHR.*
- Before we start, I’ll explain the setup and explain how the session works.

**What we’re doing today**

- Purpose/aim of today’s focus group session: we want to hear about your experiences and views of accessing and using your local community health and social care services - so your GP, pharmacy, opticians and/or dentist.
- We know this may have changed during the COVID pandemic and we want to learn more about your experiences and what you think about this topic.
- We’re trying to identify issues/problems in access to these services.
- And to do that we’re talking to people like you and based on your feedback we’re hoping to pinpoint areas for improvement.
- We have scheduled this session to run approximately x minutes.

**Room setup**

- Let me explain the setup.
- I’ll be asking you questions about your experiences and views of using your local community health and social care services - so your GP, pharmacy, opticians and/or dentist.
- No right/wrong answers

**Recording the discussion**

- We will be observing and taking notes.
- We have a microphone to record this interview for note taking purposes.
- Please remember that everything discussed in this session is confidential and do not share information outside the group.
- We may ask you for examples about how you have used digital technologies to access health services. Please don’t feel like you have to share lots of detail about the specific medical reason why you were accessing the health service.
- Housekeeping: two parts to the discussion with 10-min break. Feel free to take an informal comfort break whenever you need.
- Etiquette: don’t talk over others, let everyone speak, respect differences of opinion.
- If you wish to speak, please raise your hand. We will make sure everyone gets the chance to speak.
- Do you have any questions now before we begin?

**START RECORDING**

**---------------------------------------------------------------------------------------------------------------**

**PART A**

**Ice breaker**

- Go round room and ask each person to introduce themselves.
- What three words come to mind when you think about making appointments or using PCs, laptops or smart phones to make appointments (GP, pharmacy, optician, dentist) - it can be any three words (co-facilitator to write these down).

**INTRO**

**Experiences of using technologies to access primary care/community care?**

We would now like to ask you in more detail about your experiences of accessing primary care or community care services (such as your GP, pharmacy, optician, dentist) via digital or online? health technologies like computers, PCs, tablets or smart phones. We are interested in how this has changed during your lives, and particularly in the last three years since the start of the pandemic.

How has accessing GPs, Pharmacies etc. changed since you were younger?

- Thinking more recently, what about compared to before the pandemic? Why?
- Challenges/barriers/negative experiences?
- Facilitators/positive experiences?
- Do you feel you satisfied/listened to/confident?
- Impact/what happened as a result?
- Why/How? Age? Changes in services?
- Do you feel as though you are ‘missing out’ on anything by not using digital (online) to access primary care or community care?
- If so, what do you feel you are missing out on?
- Have you ever had any help using digital (online) technologies (PCs, smart phones, computers) (e.g., computer skills classes, from friends)
- Can you tell us about this (e.g., size of lesson/one-to-one, topics covered, length of time being helped)
- Did you find this helpful, why?
- Would you like to learn more about using the using digital (online) technology to access health services?
- What sorts of things would you like to learn?
- What would be the best way of helping you to learn more about using the Internet (e.g., class format or 1:1, topics covered, follow-up support, provision of devices?)

**---------------------------------------------------------------------------------------------------------------**

**10 MINUTE BREAK**

- Thank participants for an interesting discussion
- Take a 10-minute break
- Return at [time]

**RESUME WITH PART B QUESTIONS**

**---------------------------------------------------------------------------------------------------------------**

**PART B**

- Welcome back

We are now going to move on to the final section of the discussion, about your views and expectations about future primary care (community care) services using digital (online) health technologies.

*The first area we want to talk about is future use of technology*

- What do you think about the future for accessing GPs, pharmacies, etc. using computers etc.?
- Positives? Negatives? Why?
- Barriers/facilitators?

*The second area we want to ask about is thinking about non-technology, offline alternatives*

- Are there any health services or health needs for which you feel like it is not appropriate to use digital (online) technologies (devices, website and apps)?
- Why?
- Are there any areas of primary care (community care) services that are important to remain non-digital? (e.g., appointments, ordering prescriptions).
- Why?

*Final question*

- Before we finish, are there any other thoughts you have about the future of access to primary care (community care) services?
- Overall, how did you find the experience of accessing primary care or community care services online via websites/apps?
- Is there anything you would do differently the next time?

---------------------------------------------------------------------------------------------------------------

**CLOSE**

- Thank participants for their time and for a helpful discussion
- Details about voucher payment
- Sharing findings: we will contact participants/via the community group when the results of the study are available later this year

| 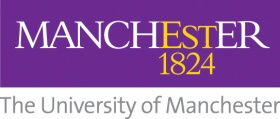 | 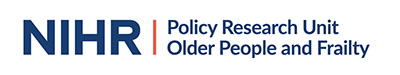 |
| --- | --- |

**Online Focus Group Topic Guide**

**Using Digital Health Technologies to Access Primary Care**

*Indicative topic guide: questions may not necessarily be asked verbatim or in the specific order they appear*

**---------------------------------------------------------------------------------------------------------------**

**INTRODUCTION**

**Welcome**

- Thank group for helping out today
- *Introduce research team and department*
- *This study is being conducted by the National Institute for Health and Care Research (NIHR) Older People and Frailty Policy Research Unit on behalf of the Department of Health and Social Care.*
- *The NIHR Older People and Frailty Policy Research Unit works to produce timely, high-quality evidence for policymakers.*
- *We are a collaboration between the University of Manchester, Newcastle University, and the London School of Economics.*
- *We answer policymakers’ questions and help the Department of Health and Social Care make informed decisions about the lives of older people.*
- *We carry out research into promoting healthy ageing, the future needs of older people and the provision of high-quality, cost-effective care.*
- *The Older People and Frailty Policy Research Unity is funded by the NIHR.*
- Before we start, I’ll explain the setup and explain how the session works.

**What we’re doing today**

- Purpose/aim of today’s focus group session: we want to hear about your experiences and views of accessing and using your local community health and social care services - so your GP, pharmacy, opticians and/or dentist.
- We know this may have changed during the COVID pandemic and we want to learn more about your experiences and what you think about this topic.
- We’re trying to identify issues/problems in access to these services.
- And to do that we’re talking to people like you and based on your feedback we’re hoping to pinpoint areas for improvement.
- We have scheduled this session to run approximately x minutes.

**Room setup**

- Let me explain the setup.
- I’ll be asking you questions about your experiences and views of accessing and using your local community health and social care services – so your GP, pharmacy, opticians and/or dentist.
- No right/wrong answers

**Recording the discussion**

- We will be observing and taking notes.
- We have a camera and a microphone to record this discussion.
- We will record the session for note taking purposes, but the footage (for online sessions) will not be seen by anyone outside the team.
- Please remember that everything discussed in this session is confidential and do not share information outside the group.
- We may ask you for examples about how you have used digital technologies to access health services. Please don’t feel like you have to share lots of detail about the specific medical reason why you were accessing the health service.
- Housekeeping: two parts to the discussion with 10-min break. Feel free to take an informal comfort break whenever you need.
- Etiquette: don’t talk over others, let everyone speak, respect differences of opinion.
- Using Zoom/Teams – raise hand function or wave.
- Do you have any questions now before we begin?

**START RECORDING**

**---------------------------------------------------------------------------------------------------------------**

**PART A**

**Ice breaker**

- Go round room and ask each person to introduce themselves
- *Occupation?*
- *Where do you live?*
- *How do you access the internet?*
- *Home? Work? Phone?*
- *Do you own a laptop or desktop?*
- *Do you have broadband?*
- *Do you use website/apps on your laptop/smartphone?*
- *What sort of website/apps do you use?*
- *Please describe*
- What three words come to mind when you think about using digital devices like computers, tablets or smartphones to make appointments with services like your GP, pharmacy, optician or dentist? (co-facilitator to write these down)

**Experiences of using technologies to access primary care**

We would now like to ask you in more detail about your experiences of accessing primary care (community care) services, such as your GP, pharmacy, optician or dentist, via digital (online) technologies like computers, tablets or smartphones.

- Can you tell us how you use digital (online) technologies to access these kinds of health care services?
- What are the specific services you use digital (online) technologies for?
- What are the specific apps you use or websites you visit for these kind of health services? Why?
- What device do you usually use – e.g., computer, laptop, iPad, tablet, smart phone? Why?
- What do you like about accessing these kind of health services through digital (online) technologies?
- What do you dislike about it?
- What, if any, concerns do you have about accessing these kind of health services through digital (online) technologies?
- Are there any other experiences you would like to share?
- When using digital technologies to access these kind of health services, do you feel you are satisfied/listened to/confident about the service you are receiving?
- Would anyone like to give us a specific example (without going into detail about the reason why you were using the service!) – which service were you trying to use, how did you use technology to do this, and what happened as a result?
- Is your experience of using digital (online) technologies to access primary care (community care) different now compared to when you were younger?
- Why/How? Age? Changes in services?
- What about compared to before the pandemic? Why?
- Challenges/barriers/negative experiences?
- Facilitators/positive experiences?

**---------------------------------------------------------------------------------------------------------------**

**10 MINUTE BREAK**

- Thank participants for an interesting discussion
- Take a 10-minute break
- Return at [time]

**RESUME WITH PART B QUESTIONS**

**---------------------------------------------------------------------------------------------------------------**

**PART B**

Welcome back

- We are now going to move on to the final section of the discussion, about your views and expectations about future primary care (community care) services using digital (online) health technologies.

*The first area we want to ask about is future use of technology*

- What do you think about the future of accessing health services like your GP, pharmacy, dentist or optician using digital (online) technologies?
- What do you think about the future for accessing GPs, pharmacies, etc. using computers etc.?
- Positives? Negatives? Why
- Barriers/facilitators?

*The second area we want to ask about is thinking about non-technology, offline alternatives*

- Are there any health services or health needs for which you feel like it is not appropriate to use digital (online) technologies (devices, website and apps)
- Why
- Are there any areas of primary care (community care) services that are important to remain non-digital? (e.g., appointments, ordering prescriptions
- Why?

*Final question*

- Before we finish, are there any other thoughts you have about the future of access to primary care (community care) services?
- Overall, how did you find the experience of accessing primary care or community care services online via websites/apps?
- Is there anything you would do differently the next time?

---------------------------------------------------------------------------------------------------------------

**CLOSE**

- Thank participants for their time and for a helpful discussion
- Details about voucher payment
- Sharing findings: we will contact participants/via the community group when the results of the study are available later this year
