# Supplementary material for: Experiences and Views of Older Adults of South Asian, Black African, and Caribbean Backgrounds About the Digitalization of Primary Care Services Since the COVID-19 Pandemic: Qualitative Focus Group Study
Source: JMIR Form Res. 2024 Dec 18;8:e57580. doi: 10.2196/57580 (PMC11656993; doi:10.2196/57580)
Supplement: Multimedia Appendix 2 [file formative_v8i1e57580_app2.docx]

**Supplementary file 1: Consolidated Criteria for Reporting Qualitative Research (COREQ): 32-item Checklist**

| **No.** | **Item** | **Guide questions/description** | **Study Details** |
| --- | --- | --- | --- |
| **Domain 1: Research team and reflexivity (Personal Characteristics)** | | | |
| **1** | Interviewer/facilitator | Which author/s conducted the interview or focus group? | The focus groups were conducted by NA and BP, supported by AH and JM. |
| **2** | Credentials | What were the researcher’s credentials? E.g. PhD, MD | NA, BP, and AH, CT, JLA (PhDs), and JM (MA). |
| **3** | Occupation | What was their occupation at the time of the study? | NA, BP, and AH (Research Fellows); CT (Director of OPFPRU & Professor of Primary Care and Community Health); JLA (Research Associate); JM (PRU Manager / PCIE Lead). |
| **4** | Gender | Was the researcher male or female? | NA (Male)  AH (Male)  BP (Female)  JM (Female)  JLA (Female)  CT (Male) |
| **5** | Experience and training | What experience or training did the researcher have? | The researchers were highly trained in mixed methods research, qualitative research techniques, and focus group facilitation. |
| **6** | **Relationship with Participants**  Relationship established | Was a relationship established prior to study commencement? | Yes, relationships were established through community groups, organisations and advisory networks prior to study commencement. |
| **7** | Participant knowledge of the interviewer | What did the participants know about the researcher? e.g. personal goals, reasons for doing the research | Participants were briefed on the research objectives and context, as well as informed about the researchers' roles in the study and their academic backgrounds. |
| **8** | Interviewer characteristics | What characteristics were reported about the interviewer/facilitator? e.g. Bias, assumptions, reasons and interests in the research topic | Facilitators shared ethnic background with participants, and their research experience and interests, no explicit biases reported. |
| **Domain 2: Study design** | | | |
| **9** | **Theoretical framework**  Methodological orientation and Theory | What methodological orientation was stated to underpin the study? e.g. grounded theory, discourse analysis, ethnography, phenomenology, content analysis | The study employed a framework analysis approach, with a focus on qualitative research. |
| **10** | **Participant selection**  Sampling | How were participants selected? e.g. purposive, convenience, consecutive, snowball | Participants were selected using purposive and convenience sampling. |
| **11** | Method of approach | How were participants approached? e.g. face-to-face, telephone, mail, email | Participants were approached via community organisations, direct recruitment and online advertisements, using face-face, telephone, and email communication. |
| **12** | Sample size | How many participants were in the study? | A total of 27 participants, of which 22 were female and 5 were male. |
| **13** | Non-participation | How many people refused to participate or dropped out? Reasons? | Non-participation details were not reported, there were no drop-outs during the study. |
| **14** | Setting of data collection | Where was the data collected? e.g. home, clinic, workplace | Focus group data were collected in community settings through face-to-face, in-person sessions and online via Zoom for virtual/online groups. |
| **15** | Presence of non-participants | Was anyone else present besides the participants and researchers? | Yes, community group organisers were present with the participants and researchers during the in-person focus group sessions, but they were not involved in the online sessions, where only researchers and participants were present. |
| **16** | Description of sample | What are the important characteristics of the sample? e.g. demographic data, date | Participants were older adults (65+) from South Asian, Black African, and Caribbean backgrounds in the UK. Participant characteristics (n=27) are presented in Table 1. Data were collected in April and May 2023. |
| **17** | **Data collection**  Interview guide | Were questions, prompts, guides provided by the authors? Was it pilot tested? | Topic guides for the focus groups were developed as structured outlines or lists of key topics, questions, and prompts to steer the discussion. These guides were designed to keep the conversation aligned with the research objectives and addressed the main themes the researchers aimed to explore. The interview guide was pilot tested, and feedback was obtained from community workers experienced in conducting focus groups within their communities. |
| **18** | Repeat interviews | Were repeat interviews carried out? If yes, how many? | No repeat interviews were conducted. |
| **19** | Audio/visual recording | Did the research use audio or visual recording to collect the data? | Yes, focus groups were recorded using Zoom (online sessions) and an encrypted audio-recording device (in-person sessions). |
| **20** | Field notes | Were field notes made during and/or after the interview or focus group? | Field notes were made by researchers during and after focus groups. |
| **21** | Duration | What was the duration of the interviews or focus group? | Focus groups lasted up to two hours, including breaks. |
| **22** | Data saturation | Was data saturation discussed? | Yes, data saturation was addressed during the analysis. The structured framework analysis approach provided a rigorous, transparent, and comprehensive examination of the qualitative data, which helped identify significant patterns related to the research questions while remaining grounded in participants' experiences. |
| **23** | Transcripts returned | Were transcripts returned to participants for comment and/or correction? | No, transcripts were not returned to participants for comment or correction. |
| **Domain 3: Analysis and findings** | | | |
| **24** | Number of data coders | How many data coders coded the data? | Two researchers (NA and AH) coded the same transcript using a broadly inductive approach, staying close to the data. |
| **25** | Description of the coding tree | Did authors provide a description of the coding tree? | Yes, the authors provided a description of the coding tree. They described the process of coding, developing an analytical framework, applying the framework, charting data, and interpreting data. This description includes how they systematically applied codes, grouped them into thematic categories, and used a framework matrix to organise and interpret the data. |
| **26** | Derivation of themes | Were themes identified in advance or derived from the data? | Themes were derived from the data through framework analysis. |
| **27** | Software | What software, if applicable, was used to manage the data? | MS Excel was used to manage the data. |
| **28** | Participant checking | Did participants provide feedback on the findings? | Participants did not provide feedback on the findings, but the results and findings will be shared with them. |
| **29** | **Reporting**  Quotations presented | Were participant quotations presented to illustrate the themes / findings? Was each quotation identified? e.g. participant number | Yes, participant quotations were used to illustrate the themes and findings, and each quotation was identified with participant numbers. |
| **30** | Data and findings consistent | Was there consistency between the data presented and the findings? | Yes, the data presented were consistent with the findings reported. |
| **31** | Clarity of major themes | Were major themes clearly presented in the findings? | Yes, major themes were clearly presented in the findings. |
| **32** | Clarity of minor themes | Is there a description of diverse cases or discussion of minor themes? | Yes, diverse cases and minor themes were discussed in the analysis. |
